# Clinical course and seizure outcome of idiopathic childhood epilepsy: determinants of early and long-term prognosis

**DOI:** 10.1186/1471-2377-13-206

**Published:** 2013-12-18

**Authors:** Pinelopi Dragoumi, Olga Tzetzi, Efthimia Vargiami, Evangelos Pavlou, Konstantinos Krikonis, Eleftherios Kontopoulos, Dimitrios I Zafeiriou

**Affiliations:** 1 Department of Pediatrics, Aristotle University of Thessaloniki, Hippokration General Hospital, Thessaloniki, Greece; 2 Department of Pediatrics, Aristotle University of Thessaloniki, AHEPA General Hospital, Thessaloniki, Greece; 3 Statistical Data Analysis, DatAnalysis, Thessaloniki, Greece

**Keywords:** Epilepsy, Idiopathic, Children, Course, Prognosis

## Abstract

**Background:**

Idiopathic epilepsies and epileptic syndromes predominate childhood and adolescence epilepsy. The aim of the present study was to investigate the clinical course and outcome of idiopathic childhood epilepsy and identify variables determining both early and long-term prognosis.

**Methods:**

We followed 303 children with newly diagnosed idiopathic epilepsy aged 1–14 years old, both prospectively and retrospectively. Outcome was defined at one, 2 and 4 years of follow-up, as well as at the end of the study period for all patients. Based on the data collected, patients were classified in four patterns of clinical course: “excellent”, “improving”, “relapsing” and “poor”. Variables defined at intake and after the initial year of treatment were analyzed for their prognostic relevance towards the clinical course and outcome of the patients.

**Results:**

The mean age at seizure onset was 6,7 years and the mean duration of follow-up was 8,3 years (range 2,0-22,0,SD 4,24). During the initial year of treatment, 70,3% of patients were seizure-free. The course of epilepsy was “excellent” in 53,1% of the subjects, “improving” in 22,8%, “relapsing” in 22,1% whereas only 6 children with idiopathic epilepsy (2%) had a “poor” clinical course exhibiting drug-resistance. After multivariate analysis, variables predictive of a poor initial response to therapy were early seizure onset, multiple seizure types and history of status epilepticus. At the end of follow-up, early response to treatment was of significant positive predictive value, while the presence of multiple seizure types and the history of migraine had a negative impact on prognosis.

**Conclusions:**

In the vast majority of children, the long-term prognosis of idiopathic epilepsy is favorable. More than half of the patients attain seizure freedom immediately and their clinical course is considered “excellent”. About one fifth exhibit either an improving or a fluctuating course. Early seizure onset, multiple seizure types and status epilepticus are predictive of an initial poor response to treatment in children with idiopathic epilepsy. Initial non-response to treatment, multiple seizure types and history of migraine are determinants of a less favorable final outcome after long-term follow-up.

## Background

Epilepsy is one of the most common chronic neurologic disorders diagnosed in children and adolescents, with an estimated prevalence in Europe of 4,5-5 per 1000 [1]. The vast majority of epilepsies encountered under the age of 15 years are idiopathic, developing without any identifiable or suspected cause, other than a genetic predisposition in many cases. A large number of children with “epilepsy-only” are attended and followed-up even by general paediatricians in everyday clinical practice. Therefore, study of the clinical course and seizure outcome of idiopathic epileptic syndromes and epilepsies is of the utmost importance in the treatment of children and adolescents with epilepsy.

It is broadly recognized that the individual prognosis of epilepsy reflects primarily its underlying etiology and syndromic classification. With respect to seizure control, studies of the natural history of new-onset childhood epilepsy indicate that epilepsies classified as idiopathic have an overall good prognosis [2,3]. However, it has been proven that the course of epilepsy and the outcome regarding seizure occurrence, both at short-term observation and after many years of follow-up, vary considerably, even among patients with the same epileptic syndrome and determinants of the individual prognosis among idiopathic childhood epilepsies remain largely unknown. Most of the existing reports on the prognosis of childhood epilepsy comprise cases of both idiopathic and non-idiopathic aetiology, including subjects with self-remitting benign epileptic syndromes and others with drug resistant symptomatic epilepsies or epileptic encephalopathies. In the present study we attempted to investigate the clinical course and outcome in a homogeneous group of children and adolescents with idiopathic epilepsy without any underlying etiology or major comorbidities, and to determine factors associated with a poor response to therapy among this subgroup of epilepsies with an overall favorable prognosis.

## Methods

### Patients

Children and adolescents under the age of 14 years old who were diagnosed with idiopathic epilepsy before December 31^st^ 2010 were included in the study. Subjects were identified on the basis of hospital and outpatient epilepsy clinic records. All were prescribed AED treatment and were followed-up until December 31, 2012. Altogether, 303 eligible consecutive patients were identified, 47 first evaluated for epilepsy between January 1^st^ 2008 and December 31^st^ 2010 and followed-up prospectively, and 256 diagnosed before January 2008 and followed both retrospectively and prospectively. For the retrospective group the diagnosis was based on sufficient and detailed clinical and EEG documentation and review of their entire records by a team of two child neurologists. Subjects were excluded from the study when matters of non-compliance to therapy were encountered, when episodes initially considered being epileptic seizures proved to be of non-epileptic nature or when the initial diagnosis of idiopathic aetiology was contradicted by newer findings. All patients were reevaluated at the end of the study follow-up, by December 31, 2012.

### Treatment

Upon diagnosis, an appropriate antiepileptic drug (AED) was chosen by a specialized child neurologist, taking into account the seizure type, syndromic classification, side-effect and interaction profiles. In principle, treatment was commenced after two or more seizures, with a single drug when possible. Patients were subsequently evaluated at the epilepsy outpatient clinic. At every follow-up visit, clinical information and the response to AED treatment were recorded. Drug doses were adjusted as clinical circumstances and weight growth dictated and treatment was altered to another drug if seizures continued using an appropriate daily dose or if the patient developed a severe adverse effect. A combination of drugs was used in children whose epilepsy remained uncontrolled despite treatment with two or three single drug regimens.

### Study protocol

According to the study protocol, early response to treatment during the initial 12 months was recorded for all patients and the outcome regarding seizure control was documented at two years of follow-up (short-term), at four years of follow-up (long-term), as well as at the end of data collection. For a minority of subjects who were enrolled to the study between January 1 2009 and December 31 2010, long-term outcome of seizure control could not be documented as yet at the time of writing of the article. The course of epilepsy was assessed by comparing the initial 12 months, second year, fourth year and last year of follow-up on the occurrence of seizures or not.

### Definitions

Seizures and epileptic syndromes were classified according to the guidelines of the International League Against Epilepsy (ILAE) [4]. The syndromic classification according to ILAE 1989 has been replaced by the new proposed diagnostic scheme in the report of ILAE task force on classification and terminology of 2006 [5,6]. Epilepsy and epileptic syndromes were classified as idiopathic depending upon factors such as age at seizure onset, type of seizures, electroencephalographic changes, family history, absence of an anatomic brain lesion or other neurological signs or symptoms and a presumed genetic origin.

In this study, “early remission” was defined as seizure-freedom achieved immediately or within the initial 3 months of treatment, whereas “initial non-response” was defined as the occurrence of seizures beyond the first 3 months of follow-up during the initial year of treatment. Patients were defined as having a “relapse” when seizures were controlled for more than 12 consecutive months and reoccurred thereafter. At the end of the two years follow-up period, patients were considered to be in “remission” if they had not had seizures of any type for a minimum of one year while receiving appropriate antiepileptic drug treatment. At the end of the four years’ follow-up, they were considered to be in “long-term remission” if they had reported no seizures for a minimum of two years. “Terminal remission” was defined as being seizure free for a minimum of 2 years preceding the end of follow-up. For those subjects who were followed prospectively the end of follow-up was considered to be the time of their last visit, whereas for those observed retrospectively the time of their reevaluation by December 2012. Patients who were never seizure free for a whole year were considered to be “drug-resistant”. All definitions were applied to patients following an appropriate antiepileptic drug treatment at the proper daily doses.

As far as the clinical course of the idiopathic epilepsy is concerned, patients were classified into four mutually exclusive patterns of clinical course, following the methodology used in a previous study of both children and adults with newly diagnosed epilepsy by Brodie, Kwan and associates [7]. In pattern A, defined as “excellent” clinical course, children became seizure free early and remained so throughout follow-up. In pattern B, seizure freedom was delayed for more than 12 months after starting treatment but patients remained seizure free throughout their follow up and thus this clinical course was defined as “improving”. Patients with pattern C were considered as having a “relapsing” clinical course, with periods of seizure freedom lasting more than one year interspersed with relapses. Finally, patients exhibiting pattern D, defined as “poor” clinical course, were “drug-resistant” throughout follow-up, as explained above.

### Variables and investigations

A priori defined variables based on individual and clinical characteristics of each patient, “seizure” history, EEG and neuroimaging findings, as well as history of coexisting migraine or sleep disorders were collected at intake and after 12 months of treatment. *Status epilepticus* was defined as continuous seizure activity or not regaining consciousness between seizures for at least 30 minutes [8]. *Academic performance* was evaluated on the basis of family and teachers’ information and no objective screening tool was applied to the subjects of the present study. It was considered “excellent” when children received high grades in school, “good” when they followed lessons without any difficulty, “average” when they encountered minor difficulties and “poor” when they had significant difficulty or learning disorders. All children had a *standard electroencephalogram (EEG)*. In children without epileptiform abnormalities, a second EEG was performed after partial sleep deprivation. *Neuroimaging* was performed with magnetic resonance imaging (MRI) or computed tomography (CT) when the treating physician considered it reevaluated at the end of the study follow-up, by December 31, 2012 necessary.

### Statistical analyses

Data were analyzed using the IBM SPSS for Windows v.20 software (IBM, New York, USA). Numerical variables were presented as mean (±SD) except for non-normal distributed variables, where the median, interquartile range (IQR), and range was calculated. Kolmogorov-Smirnov test was used to evaluate whether each variable followed a Gaussian distribution. Categorical variables were summarized using frequencies and percentages. The relationships among study variables were investigated using Pearson product–moment correlation coefficient (r), whereas correlations including at least one non-normal variable were performed using Spearman correlation coefficient (rho). Categorical variable association was assessed using chi-square test or Fisher’s exact test when appropriate. We used binary logistic regression to study the predictors of terminal remission. Multivariate logistic regression was used to evaluate the combined effect of the strongest predictors regarding early, two year and long term outcome. Model calibration was evaluated using the Hosmer-Lemeshow goodness-of-fit test. In the univariate analysis preceding the multivariate, potential prognostic factors were assessed using P-value <0.05 as a cut-off criterion. In the multivariate analysis backward elimination stepwise procedure was used, where variables having p-value greater than 0.10 were removed from the models. The probability curves of terminal remission were obtained according to the Kaplan-Meier method and compared by the log-rank test.

## Results

A total of 303 children and adolescents (N = 160, 52,8% male, N = 143, 47,2% female), diagnosed with epilepsy of idiopathic etiology were included in the study. The mean age at onset of epilepsy was 6,7 years (median 6,5, range 1,0-14,0, std. dev. 3,0) and the mean duration of follow-up was 8,3 years (median 7,0, range 2,0-22,0, std. dev. 4,24). In 255 of 303 subjects a specific idiopathic epileptic syndrome was recognized, while in the remaining 48 (15,8%) epilepsy was considered as being idiopathic generalized (N = 14, 4,6%), idiopathic partial (N = 31, 10,2%) or idiopathic otherwise unclassified (N = 3, 1,0%).

Table 1 summarizes the clinical features of the study population. 18,5% of the children and adolescents with idiopathic epilepsy (N = 56/303) had a history of febrile seizures, 21,2% (N = 64/303) a positive family history of epilepsy and 16,2% (N = 49/303) a history of concomitant migraine disorder. 8,3% of the subjects (N = 25/303) were documented as having sleep disorders. Regarding their academic performance, the majority of children (61,7%) accomplished all school requirements without any difficulty (“good” academic performance), 6,3% (N = 19) had an “excellent” performance, 21,5% (N = 65) encountered mild difficulties in the learning procedure (“average” academic performance) and 10.6% (N = 32) exhibited a “poor” academic performance with significant learning disorders. Regarding their seizure activity, 12,5% of the children (N = 38) manifested more than one seizure types and only 3,3% (N = 10) were documented as having had status epilepticus. EEG findings were normal in 9,6% of the patients, focal in 50,8%, generalized in 44,2%, while in 4% (N = 12) of the children background activity exhibited generalized slowing. Neuroimaging studies were performed in 216 children and adolescents, with normal findings in 206 (95,4%) of the cases. Minor abnormalities, not causally correlated with seizures and epilepsy, were reported in 10 of the cases (3,3%).

**Table 1:** Characteristics of the study population

	**N**	**%**
**Sex**		
Male	160	52,8
Female	143	47,2
**Age at seizure onset**		
1-4 years	86	28,4
>4-6 years	65	21,5
>6-9 years	89	29,4
>9-12 years	55	18,2
>12 years old	8	2,6
**History of febrile seizures**		
No	247	81,5
Yes	56	18,5
**Family history of seizures**		
No	239	78.9
Yes	64	21,1
**History of migraine**		
No	254	83,8
Yes	49	16,2
**Sleep disorders**		
No	278	91,7
Yes	25	8,3
**Academic performance**		
Poor	32	10,6
Average	65	21,5
Good	187	61,7
Excellent	19	6,3
**Time elapsed between seizure onset and treatment with AEDs**		
No time	81	26,7
6 months	170	56,1
6-12 months	19	6,3
>1 year	33	10,9
**Number of episodes before onset of treatment**		
1-2 episodes	118	38,9
3-5 episodes	81	26,7
>5 episodes	104	34,3
**Number of seizure types**		
One	265	87,5
More than one	38	12,5
**Status epilepticus**		
No	293	96,7
Yes	10	3,3
**EEG findings**		
Normal	29	9,6
Abnormal background activity	12	4,0
Focal discharges	154	50,8
Generalized discharges	134	44,2
**Neuroimaging**		
Normal	206	68,0
Abnormal	10	3,3
Not conducted	87	28,7

### Early response to treatment

During the initial 12 months of follow-up, “early remission” was observed in 213 patients (70,3%) of the cohort, while the remaining 90 patients (29,7%) exhibited seizure activity beyond the initial 3 months of follow-up. 53 patients (17,5%) had less than 3 epileptic episodes, 27 patients (8,9%) more than 3 but less than 6 episodes and only 10 patients (3,3%) had frequent seizures (>6 episodes/year).

### Final outcome (outcome at last follow-up)

**Figure 1:**
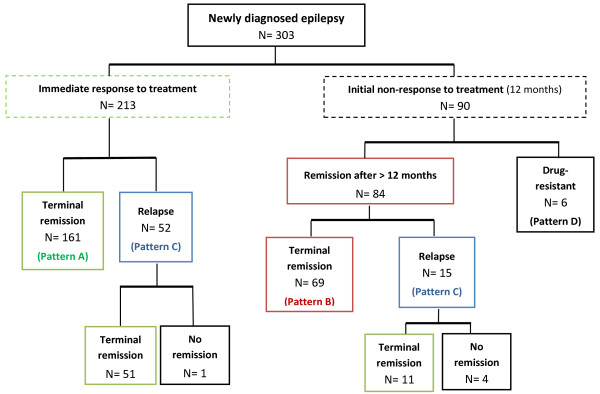
**Patient flow throughout the study in terms of seizure outcome.** Terminal remission: seizure-freedom of at least 2 years preceding the end of follow-up. Pattern A (early and sustained seizure freedom): patients became seizure-free within 12 months of starting treatment and remained seizure-free. Pattern B (delayed and sustained seizure freedom): patients became seizure-free after 12 months of starting treatment and remained seizure-free. Pattern C (fluctuating course): patients fluctuating between periods of seizure freedom and relapse. Pattern D: patients never seizure-free for any complete year.

At the end of the study, a total of 292 subjects (96,3%) were in terminal remission (N = 251, 82,8% off AEDs, N = 41, 13,5% on AEDs). Epilepsy was still active in the remaining 11 subjects (3,7%), 6 of whom were intractable, with no remission exceeding 12 months during follow-up, and 5 were in relapse at the end of follow-up. Table 2 summarizes the clinical course and outcome of each epilepsy syndrome and Figure 1 shows the patients’ flow throughout the study in terms of seizure outcome.

**Table 2:** Remission, intractability and course of epilepsy during follow-up for each idiopathic epilepsy type and syndrome (ILAE)

		**Remission**	**No remission**	**“Excellent” course**	**“Improving” course**	**“Relapsing” course**	**“Poor” course**
	**N**	**N (%)**	**N (%)**	**N (%)**	**N (%)**	**N (%)**	**N (%)**
Benign childhood epilepsy with centrotemporal spikes	107	107 (100,0)	0	61 (57,0)	22 (20,5)	24 (22,5)	0
Early onset benign occipital epilepsy (Panayiotopoulos type)	5	5 (100,0)	0	2 (40,0)	3 (60,0)	0	0
Late onset occipital epilepsy (Gastaut type)	2	2 (100,0)	0	1 (50,0)	0	1 (50,0)	0
Benign epilepsy of infancy	2	2 (100,0)	0	1 (50,0)	1 (50,0)	0	0
Myoclonic epilepsy in infancy	3	3 (100,0)	0	1 (33,3)	1 (33,3)	1 (33,3)	0
Childhood absence epilepsy	80	70 (98,7)	1 (1,3)	53 (66,2)	15 (18,8)	11 (13,7)	1 (1,3)
Juvenile absence epilepsy	5	5 (100,0)	0	4 (80,0)	0	1 (20,0)	0
Juvenile myoclonic epilepsy	5	4 (80,0)	1 (20,0)	1 (20,0)	0	4 (80,0)	0
Epilepsy with GTCS only	16	16 (100,0)	0	9 (56,3)	3 (18,7)	4 (25,0)	0
Generalized epilepsies with febrile seizures +	9	9 (100,0)	0	5 (55,6)	0	4 (44,4)	0
Epilepsy with myoclonic-astatic seizures	8	7 (87,5)	1 (12,5)	0	6 (75,0)	1 (12,5)	1 (12,5)
Epilepsy with myoclonic absences	5	5 (100,0)	0	0	2 (40,0)	3 (60,0)	0
Idiopathic partial epilepsy	31	26 (83,8)	5 (16,2)	11 (35,5)	11 (35,5)	5 (16,1)	4 (12,9)
Idiopathic generalized epilepsy	14	13 (92,8)	1 (7,2)	9 (64,3)	3 (21,4)	2 (14,2)	0
Photosensitive-reflex epilepsy	8	7 (87,5)	1 (12,5)	3 (37,5)	1 (12,5)	4 (50,0)	0
Idiopathic unclassified	3	2 (66,7)	1 (33,3)	0	1 (33,3)	2 (66,7)	0
Total	303	292 (96,3)	11 (3,7)	161 (53,1)	69 (22,8)	67 (22,1)	6 (2,0)

### AED treatment

Among the group of patients who achieved terminal remission, a total of 244 (83,6%) remained seizure-free on their first AED, while 36 (12,3%) were treated with a second AED, as monotherapy or in combination with the first drug. Only 11 patients (3,7%) with idiopathic epilepsy attained seizure freedom receiving 3 AEDs and 1 (0,4%) requiring >3 AEDs.

### Epileptic syndrome

The two most frequently encountered idiopathic epileptic syndromes in our study were benign childhood epilepsy with centrotemporal spikes (BCECTS) and childhood absence epilepsy (CAE). 107 patients (35,3%) were diagnosed with BCECTS, the ratio of boys to girls exceeding 3/2, with a mean age at seizure onset of approximately 7 years (peak at 4 years for boys and 8 years for girls). CAE was diagnosed in 80 patients (26,4%) of the cohort, mostly girls, with a ratio of 3/2 versus boys, and a mean age at seizure onset of 6,4 years for boys and 5,9 for girls (peak at 4 years for both sexes). Statistical analysis of their clinical characteristics and prognosis demonstrated the following: (a) regarding clinical characteristics, there was a higher incidence of poor – average *academic performance* documented in children with CAE as opposed to children with rolandic epilepsy (p = 0,011), (b) regarding clinical course and seizure outcome, there was no notable difference in the early response to treatment, long-term outcome, final outcome and clinical course of epilepsy between the two syndromes, however the *short-term outcome after 2 years of follow-up* was significantly more favorable (p = 0,01) in children with absence epilepsy as opposed to those treated for rolandic epilepsy.

Regarding the prognosis of idiopathic epileptic syndromes other than CAE and BCECTS, as presented in Table 2, all patients with Panayiotopoulos syndrome (N = 5/5) and Benign epilepsy in infancy (N = 2/2) achieved terminal remission following a benign non-relapsing clinical course. Both patients diagnosed with Gastaut type occipital epilepsy and Myoclonic epilepsy in infancy also achieved seizure control at final follow-up, but one or more relapses were noted in the course of epilepsy of single subjects. Patients with Juvenile myoclonic epilepsy exhibited the highest rate of a relapsing course (80%), followed by Epilepsy with myoclonic absences (60%) and Photosensitive-reflex epilepsy (50%). Finally, the highest rate of drug-resistance was found among children diagnosed with Epilepsy with myoclonic-astatic seizures (12,5%) and Idiopathic partial epilepsy otherwise not classified (12,9%).

### Course during follow-up

An “excellent” clinical course, or pattern A, was observed in 161 children and adolescents with idiopathic epilepsy (53,1%), who became seizure-free immediately and remained so for the remainder of the follow-up. Among these 161 patients, 157 (97,5%) received 1 AED and only 4 patients (2,5%) were given 2. None of the subjects with outcome pattern A required 3 or more AEDs during follow-up.

Outcome pattern B, considered to represent an “improving” clinical course, was identified in 69 patients (22,8%), who continued to report seizures during the first 12 months of treatment, but later became and remained seizure-free until the end of follow-up. More than half of these patients (N = 39, 56,5%) achieved seizure freedom treated with their first AED, 22 (31,9%) required a second AED and 8 (11,6%) required a third one.

A total of 67 patients (22,1%) demonstrated a more fluctuating course (outcome pattern C). After attaining a seizure-free period of a year or more, they were reported to have a relapse, either being on AEDs or after discontinuation of treatment. 51 out of 67 (76%) subjects following a remitting-relapsing course received 1 AED, 12 patients (18%) received 2 and 4 (5%) required treatment with 3 or more AEDs.

Only 6 children and adolescents with idiopathic epilepsy (2%) never became seizure-free for any complete year throughout follow-up (pattern D), representing the subgroup of patient population with a “poor” clinical course. Among these 6 patients, 3 received 2 regimens, 1 received 3 and the remaining 2 were treated with more than 3 AEDs.

### Proportion of seizure remission during follow-up

**Figure 2:**
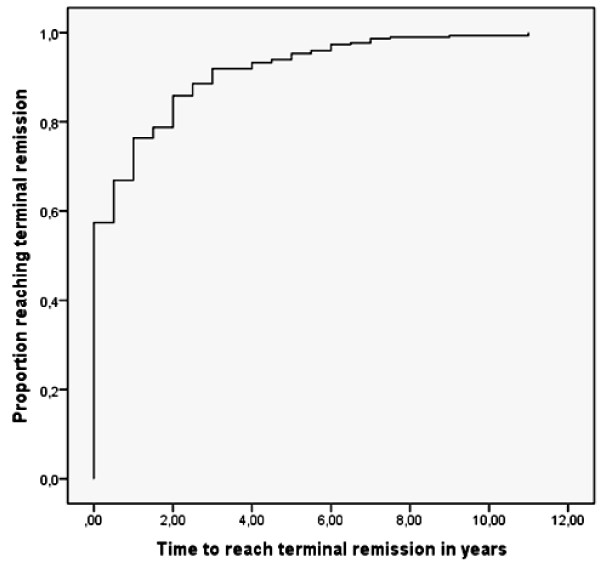
**Kaplan-Meier curve indicating the cumulative proportion of study subjects reaching terminal remission during follow-up.** Terminal remission: seizure-freedom of at least 2 years.

Figure 2 shows the proportion of subjects reaching terminal remission during the years of follow-up. As demonstrated, after 2 years of follow-up almost 80% of children with idiopathic epilepsy have the chance to achieve remission of seizures. After 4, 6, 8 and 10 years of follow-up, the chance of remission reaches 90%, 96%, 98% and 99% of the patients.

### Predictive variables

The determinants of early and long-term seizure outcome and clinical course of idiopathic childhood epilepsy after multivariate analysis are summarized in Table 3. An additional table available as supplementary material provides the results of the univariate analysis [see Additional file 1]. After both univariate and multivariate analysis, *younger age at seizure onset* was strongly associated with initial non-response to treatment (p = 0,003) and a “relapsing“ course of epilepsy (p = 0,004), whereas it didn’t appear to determine final seizure outcome at the end of follow-up. The same relevance applied to the occurrence of *status epilepticus* (p = 0,045) and positive *history of febrile seizures* (p = 0,008) after univariate analysis, but their statistical relevance subsided after multivariate analysis. On the other hand, the presence of *multiple seizure types* and *history of migraine* were strongly associated with an unfavorable prognosis both after univariate and multivariate analysis. Multiple seizure types were prognostic of a poorer outcome during the initial year of treatment (p = 0,000), and a “relapsing” overall clinical course (p = 0,004). History or migraine was associated with a decreased chance of remission after long-term follow-up (p = 0,002), as well as at the end of follow-up (p = 0,0,14) and furthermore associated with an increased chance of a “relapsing” clinical course (p = 0,03).

**Table 3:** Multivariate analysis of prognostic factors

**Prognostic factors**		***p-value***	***OR***	***95% CI***
**Early response to treatment – occurrence of seizures in the initial 12 months**				
*Associated with an increased chance of seizure occurrence*				
Multiple seizure types		0,000	3,905	1,897-8,035
Status epilepticus		0,051	3,769	0,997-14,249
*Associated with a decreased chance of seizure occurrence*				
Age at seizure onset		0,003	0,707	0,561-0,889
**Outcome at 2 years of follow-up – remission of seizures**				
*Associated with an increased chance of remission*				
Epileptic syndrome (CAE)		0,009	3,174	1,329-7,579
Early response to treatment		0,000	3,291	1,795-6,034
**Outcome at 4 years of follow-up – occurrence of seizures in the preceding two years**				
*Associated with an increased chance of seizure occurrence*				
History of febrile seizures		0,011	2,788	1,271-6,116
History of migraine		0,002	3,446	1,571-7,559
*Associated with a decreased chance of seizure occurrence*				
Academic performance		0,056	0,651	0,419-1,010
**Final outcome at the end of follow-up – occurrence of seizures in the preceding two years**				
*Associated with an increased chance of seizure occurrence*				
History of migraine		0,014	5,305	1,393-20,193
Multiple seizure types		0,069	3,413	0,911-12,789
Initial non response to treatment		0,004	10,95	2,18-54,99
**Clinical course – relapsing pattern C**				
*Associated with an increased chance of pattern C-“relapsing” clinical course*				
Multiple seizure types		0,004	3,308	1,449-7,547
History of migraine		0,03	2,309	1,083-4,922
*Associated with a decreased chance of pattern C -“relapsing” clinical course*				
Early response to treatment		0,013	0,395	0,191-0,820
Age at seizure onset		0,004	0,671	0,511-0,879

OR (odds ratio).

CAE (childhood absence epilepsy).

The most important predictive factor of the clinical course and final outcome of idiopathic epilepsy after multivariate analysis was the *initial response to antiepileptic treatment*. Early response to treatment was strongly correlated with remission after short-term follow-up (p = 0,000) with a decreased chance of following a relapsing course (p = 0,013). Children with initial non response to treatment carried a 10-fold higher risk of not reaching terminal remission at the end of follow-up (p = 0,004, OR = 10,95).

**Figure 3:**
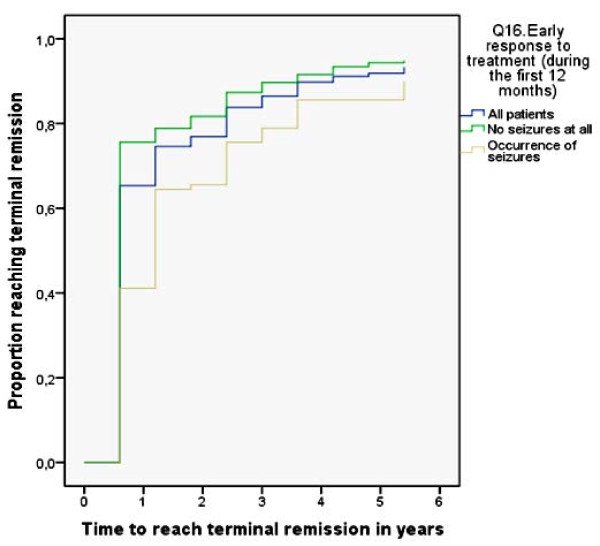
**Kaplan-Meier curve indicating the cumulative proportion of study subjects reaching 6-month remission during follow-up according to initial response to treatment.** The green line represents the patients who were seizure-free during the initial year of treatment, the yellow line the patients who experienced seizure activity and the blue line represents the total of all patients.

Figure 3 demonstrates the effect of initial response to treatment to the cumulative proportion of patients reaching a 6-month remission during follow-up. As demonstrated, after 2 years of follow-up the patients who were seizure-free during the initial year of treatment had >80% chance of being in remission, whereas the patients who exhibited seizure activity a chance of approximately 67%. After 4 years of follow-up, the chances were estimated at 92% and 86% accordingly for each of the two groups.

## Discussion

Idiopathic epilepsies and epileptic syndromes predominate childhood and adolescence epilepsy. They are age-dependent epilepsies of presumably genetic origin with an overall favorable prognosis with regard to seizure control. The aim of the present study was to observe and describe a large population of children and adolescents with idiopathic epilepsy over time in order to report the clinical course and prognosis of each epileptic syndrome and to identify variables prognostic of a less than favorable outcome after short-term and long-term follow-up.

In the available literature of the previous two decades, the prognosis of childhood epilepsy has been addressed in various studies following large cohorts of children, both retrospectively and prospectively, the duration of follow-up ranging from months to 37 years [9-15]. The majority of these studies concern children with both idiopathic and non-idiopathic epilepsies and conclude that the long-term outcome in childhood-onset epilepsy is favorable in about two-thirds of children [15]. However, when epilepsies of solely idiopathic etiology are concerned, the remission rate is considerably higher [13], reaching nearly 100% for benign childhood epilepsy with centrotemporal spikes by midadolescence [16].

In our study, after observing and analyzing the clinical course of a relatively large cohort of children and adolescents with idiopathic epileptic syndromes, the vast majority of the children had a favorable outcome at last contact, 70% of the children became immediately seizure free and after two years of follow-up the rate of remission was almost 80%. In the Dutch Study of Epilepsy in Childhood, 69% of 466 children with epilepsy were in remission after 2 years of follow-up and 71% were in long-term remission after 15 years. When referring to epileptic syndromes of idiopathic etiology, the rate of remission after 2 years of follow-up was 78% (184/235) and the rate of a 5 years terminal remission at last follow-up was 86% [15,17,18]. The lower percentage of terminal remission in the Dutch study possibly reflects the lower number of children with benign rolandic epilepsy included in the study population (23/210) as compared to the present study (107/303), given the excellent prognosis of the syndrome. In the Finish population-based study of 144 children with a mean follow-up of 37 years, Sillanpaa and Schmidt reported a five years terminal remission of 67% for both idiopathic and symptomatic epilepsies with childhood onset, which rose to 89% regarding idiopathic epilepsies, however the prognosis of specific idiopathic epileptic syndromes was not addressed [10,13].

Given the overall favorable outcome of idiopathic childhood epilepsy concluded by the available literature, the focus of research is aimed at studying the clinical course over time in order to identify determinants of early and permanent seizure control (“smooth sailing” epilepsy, [11]) as opposed to following a remitting-relapsing clinical course, as well as determinants of exhibiting a less than favorable outcome in certain epileptic syndromes and groups of patients.

In the present study, concerning children with idiopathic epilepsy, that is epilepsy only, without any underlying etiology or major comorbidities, more than half of the children had an “excellent” clinical course, with immediate response to treatment that was sustained until the end of follow-up, while an additional 23% of the cohort exhibited a delayed response, after at least 12 months of treatment, but remained seizure-free throughout follow-up. Reviewing the patterns of treatment response in newly diagnosed epilepsy studied by Brodie, Kwan and associates in a vast population of adult patients and children older than 9 years old with epilepsy, “excellent” clinical course was documented in considerably fewer patients, that is 37% of the cohort, and drug-resistance in considerably more than the present study, that is 25% as opposed to 2% [7]. These differences confirm the presence a more favorable clinical course demonstrated in childhood and epilepsies of idiopathic etiology.

Regarding the rate of a “remitting-relapsing” course in childhood epilepsy, our findings are comparable with the results of the prospective study measuring remission and relapse in children with epilepsy conducted by Berg, Shinnar and colleagues [14]. After achieving a 2-year remission, 107 out of 442 children of their cohort (24,2%) experienced a relapse and the risk of relapse varied by epileptic syndrome, with juvenile myoclonic epilepsy carrying a very high risk and idiopathic partial epilepsy a very low risk. Accordingly, in our study, the highest rates of relapse were exhibited in children with juvenile myoclonic epilepsy and epilepsy with myoclonic absences. However, when comparing the most frequent epileptic syndromes of idiopathic partial and generalized epilepsy, that is rolandic and childhood absence epilepsy, our findings demonstrate a slightly higher relapse rate experienced by children with rolandic epilepsy (22,5%) than children with CAE (13,7%), both reattaining remission after long-term follow-up. Further analysis of our study population results reveals that children diagnosed with BCECTS at a pre-school age had an increased risk of following a relapsing clinical course, without affecting their chances of terminal remission, as seen also in the study by You, Kim and Ko of Seoul, South Korea [19].

Overall comparison of the clinical characteristics and clinical course of BCECTS and CAE confirmed the use of the term “benign”, commonly assigned to both epileptic syndromes, as far as seizure control is concerned. Early response to treatment during the initial 12 months and final outcome were equally favorable among children with either epileptic syndrome, however after two years of follow-up, more children with CAE exhibited a terminal remission compared to children with BCECTS. Our findings are in agreement with those reported by Berg and associates, who found that after two years of follow-up, the highest rate of remission was exhibited by children who were documented to have typical absence seizures as compared to any other seizure type [14]. However, in terms of academic performance and cognitive outcome, a less favorable prognosis of CAE was documented in our study, in accordance with what is known from review of the current literature [20-22].

Regarding other epileptic syndromes, in the present study we encountered an invariably favorable prognosis among children with Panayiotopoulos syndrome, a high relapsing rate among patients with juvenile myoclonic epilepsy, epilepsy with myoclonic absences and photosensitive-reflex epilepsies and a considerable rate of drug resistance among children with myoclonic astatic epilepsy and idiopathic partial epilepsy not otherwise classified. However, the small number of patients allocated to each separate epileptic syndrome would not allow for safe conclusions to be drawn on their overall prognosis.

As far as predictive variables of idiopathic childhood epilepsy are concerned, multivariate analysis revealed that *multiple seizure types,* occurrence of *status epilepticus* and *young age at seizure onset* were significantly associated with a poorer initial response to treatment, while age at seizure onset between 6–9 years old was associated with an increased chance of early remission. At the end of follow-up, the single most important prognostic factor was *early response to treatment during the first 12 months*, which was highly predictive of a terminal remission at the end of follow-up. On the contrary, a *positive history of migraine* and the presence of *multiple seizures* were associated with a less favorable final outcome.

Very young age at seizure onset was found to be associated with a poorer prognosis in various studies of childhood epilepsy prognosis [9,12,18,23] and this finding can be attributed mostly to the higher prevalence of symptomatic and cryptogenic epilepsies in children during the first year of life. When focusing on idiopathic epilepsies, the distribution of specific benign epileptic syndromes at certain ages, for instance occurrence of idiopathic partial epilepsy between 6–12 years old, possibly explains the favorable prognosis experienced by children of this age group. Furthermore, even within the same epileptic syndrome, for instance BCECTS, younger age at seizure onset is found to be predictive of a poorer initial response to treatment [16,19,24].

The most significant determinant of seizure outcome in idiopathic childhood epilepsy revealed by the present study was the initial response to treatment during the first 12 months. The significance of early response to treatment as a prognostic factor was highlighted in various prognostic studies. Sillanpaa, Shinnar and colleagues reported at the New England Journal of Medicine in 1998 that the single best predictor of remission both in their overall cohort and in the subgroups classified according to etiology was an early response to drug therapy [13], while Geerts, Arts and associates, conducting the Dutch study of epilepsy in childhood, concluded that after long-term follow-up, epilepsy remained active or intractable often after having an early unfavorable course or non-idiopathic etiology [15].

Two separate clinical characteristics that were revealed in the present study as determinants of seizure outcome were the *presence of multiple seizure types* and the *positive history of migraine*. Regarding seizure types, it was shown that the occurrence of more than one seizure type was associated with a poorer initial response to treatment, as well as a less than favorable final outcome and a higher chance of a fluctuating course of epilepsy. These findings can be attributed to the coexistence of multiple seizure types in certain epileptic syndromes that exhibited a higher rate of drug-resistance or relapsing course, as for instance epilepsy with myoclonic-astatic seizures or epilepsy with myoclonic absence seizures. Furthermore, as reported in previous studies, the occurrence of generalized tonic-clonic seizures in addition to absence or myoclonic seizures is considered to be predictive of a less than favorable outcome in idiopathic generalized epilepsies of childhood and adolescence [25].

The prognostic significance of migraine disorder as a comorbidity of childhood idiopathic epilepsy has not been previously reported, as to our knowledge. While there is a vast literature reporting the similar molecular and genetic basis of the two conditions, both considered as disorders that lie in the spectrum of channelopathies [25-28], the impact of migraine on the clinical course and outcome of epilepsy has only been documented sparsely in adult epilepsy. In one prospective study published in 2005 at Cephalalgia, 59 adults with epilepsy and migraine were followed for 5–10 years and compared with 56 patients with epilepsy only and the authors concluded that the positive history of migraine disorder was associated with a decreased chance of immediate response to antiepileptic therapy, a less than favorable clinical course and a decreased chance of achieving terminal remission, especially during the last two years of the follow-up [29]. Further study is needed in order to support the negative impact of migraine disorder on the outcome of idiopathic epilepsy in childhood.

### Advantages and limitations

The size of the cohort and the length of follow-up regarding exclusively children with idiopathic epileptic syndromes are advantages of the present study. Not many cohorts are comparable on these points. The main limitation of this study is the fact that the course of epilepsy in the majority of subjects was followed retrospectively based on hospital records, a fact that initially makes proper syndromic diagnosis challenging. However, the existence of detailed clinical and EEG documentation for all retrospective subjects included in the study allowed for a syndromic classification to be safely reached in the majority of cases. Moreover, all patients were reevaluated at the end of the long-term follow-up and their clinical course and seizure outcome was accurately recorded.

## Conclusions

The long-term prognosis of idiopathic epilepsy in childhood is favorable in the vast majority of children. In terms of seizure control, the two most frequently encountered idiopathic epileptic syndromes, benign childhood epilepsy with centrotemporal spikes and childhood absence epilepsy, exhibit an excellent prognosis and can be considered as “benign”. During follow-up, the course of idiopathic childhood epilepsy is characterized by the presence of relapses in 22% of the children; however the vast majority achieve remission at the end of long-term follow-up. Predictors of a “smooth-sailing” clinical course were shown to be early response to antiepileptic drug therapy, as well as age at seizure onset between 6–9 years old. On the contrary, clinical variables such as multiple seizure types, young age at seizure onset and history of migraine were associated with a poorer prognosis.

### Ethical committee approval

xThe study was accordingly approved by the Aristotle University of Thessaloniki Bioethical Committee.

### Consent

All parents or guardians of patients entered in the study gave written informed consent prior to their entrance.

## Abbreviations

AED: Antiepileptic drug; ILAE: International League Against Epilepsy; EEG: Electroencephalogram; MRI: Magnetic resonance imaging; CT: Computed tomography; IQR: Interquartile range; BCECTS: Benign childhood epilepsy with centrotemporal spikes; CAE: Childhood absence epilepsy.

## Competing interests

The authors declare that they have no competing interests.

## Authors’ contributions

EK, DIZ, PD conceived of the study and participated in its design and coordination. EK, DIZ, OT, EV, EP and PD contributed to the acquisition of data. KK performed the statistical analysis. PD collected, interpreted and analyzed the data and drafted the manuscript. DIZ critically revised the manuscript. All authors read and approved the final manuscript.

## Pre-publication history

The pre-publication history for this paper can be accessed here:

http://www.biomedcentral.com/1471-2377/13/206/prepub

## Supplementary Material

Additional file 1 **Course and outcome of idiopathic childhood epilepsy.** Bivariate associations for prognosis of idiopathic epilepsy after univariate analysis. A table summarizing the results of the univariate analysis of prognostic factors. Click here for file
