# Supplementary material for: Clinical course and seizure outcome of idiopathic childhood epilepsy: determinants of early and long-term prognosis
Source: BMC Neurol. 2013 Dec 18;13:206. doi: 10.1186/1471-2377-13-206 (PMC3878358; doi:10.1186/1471-2377-13-206)
Supplement: Additional file 1 — Course and outcome of idiopathic childhood epilepsy. Bivariate associations for prognosis of idiopathic epilepsy after univariate analysis. A table summarizing the results of the univariate analysis of prognostic factors. [file 1471-2377-13-206-S1.pdf]

Bivariate associations for prognosis of idiopathic epilepsy after univariate analysis

Prognostic factor	N (%)	Early seizure control		Remission by 2 years FU		Remission by 4 years FU		Terminal remission		"Non-relapsing" course	
		N (%)	*p-value*	N (%)	*p-value*	N (%)	*p-value*	N (%)	*p-value*	N (%)	*p-value*
**Age at seizure onset**											
1-4 yr	86 (28)	47 (55)	*0,001 (trend)*	60 (70)		63 (77)		84 (97)		52 (60)	*0,019 (trend)*
>4-6 yr	65 (22)	43 (66)		55 (85)		52 (92)		62 (95)		55 (85)	
>6-9 yr	89 (29)	77 (87)		76 (85)		77 (92)		87 (97)		77 (86)	
>9-12 yr	55 (18)	41 (74)		44 (80)		44 (83)		52 (94)		41 (75)	
>12 yrs	8 (3)	5 (62)		6 (75)		8 (100)		7 (88)		5 (62)	
**Febrile seizures**											
no	247 (82)	182 (74)	*0,008*	200 (80)		205 (90)	*0,007*	238 (96)		192 (78)	
yes	56 (18)	31 (55)		41 (73)		39 (75)		54 (96)		38 (68)	
**Seizure type**											
one	265 (88)	197 (74)	*0,000*	220 (83)	*0,000*	217 (89)	*0,01*	259 (98)	*0,003*	210 (79)	*0,003*
more than one	38 (12)	16 (42)		21 (55)		27 (73)		33 (87)		20 (53)	
**Status epilepticus**											
no	293 (97)	209 (71)	*0,045*	233 (80)		235 (86)		282 (96)		222 (76)	
yes	10 (3)	4 (40)		8 (80)		9 (90)		10 (100)		8 (80)	
**Sleep disorders**											
no	278 (92)	200 (72)	*0,041*	223 (80)		228 (88)	*0,049*	270 (97)	*0,032*	*214 (77)*	
yes	25 (8)	13 (52)		18 (81)		16 (72)		22 (88)		*16 (64)*	
**Migraine**											
no	254 (84)	178 (70)		201 (79)		210 (90)	*0,001*	248 (97)	*0,014*	*200 (79)*	*0,033*
yes	49 (16)	35 (71)		40 (87)		34 (70)		44 (89)		*30 (61)*	
**Academic performance**											
poor	32 (11)	20 (62)		25 (78)		23 (74)	*0,012 (trend)*	28 (87)		21 (66)	
average	65 (21)	42 (65)		50 (77)		51 (82)		62 (95)		45 (69)	
good	187 (62)	139 (74)		151 (80)		153 (90)		185 (98)		148 (79)	
excellent	19 (6)	12 (63)		15 (79)		17 (90)		17 (89)		16 (84)	
**EEG**											
background slowing	12 (4)	6 (50)		6 (50)	*0,016*	11 (92)		11 (92)		7 (58)	
**Initial response to treatment**											
no seizures	213 (70)	-		185 (87)	*0,000*	173 (89)		211 (99)	*0,000*	160 (75)	
occurrence of seizures	90 (30)			56 (62)		71 (83)		81 (90)		70 (77)	
